# Hypopituitarism after subarachnoid haemorrhage, do we know enough?

**DOI:** 10.1186/s12883-014-0205-0

**Published:** 2014-10-14

**Authors:** Ladbon Khajeh, Karin Blijdorp, Sebastian JCMM Neggers, Gerard M Ribbers, Diederik WJ Dippel, Fop van Kooten

**Affiliations:** Department of Neurology, Erasmus MC University Medical Centre, P.O. Box 2040, 3000 CA Rotterdam, the Netherlands; Department of Internal Medicine, Erasmus MC University Medical Centre, Rotterdam, the Netherlands; Department of Rehabilitation Medicine, Erasmus MC University Medical Centre and Rijndam Rehabilitation Centre, Rotterdam, the Netherlands

**Keywords:** Subarachnoid haemorrhage, Hypopituitarism, Neuroendocrine, Functional outcome

## Abstract

**Background:**

Fatigue, slowness, apathy and decrease in level of activity are common long-term complaints after a subarachnoid haemorrhage (SAH). They resemble the symptoms frequently found in patients with endocrine dysfunction. Pituitary dysfunction may be the result of SAH or its complications. We therefore hypothesized that it may explain some of the long-term complaints after SAH.

We reviewed the literature to clarify the occurrence, pattern and severity of endocrine abnormalities and we attempted to identify risk factors for hypopituitarism after SAH. We also assessed the effect of hypopituitarism on long-term functional recovery after SAH.

**Methods:**

In a MEDLINE search for studies published between 1995 and 2014, we used the term subarachnoid haemorrhage in combination with pituitary, hypopituitarism, growth hormone, gonadotropin, testosterone, cortisol function, thyroid function and diabetes insipidus. We selected all case-series and cohort studies reporting endocrine function at least 3 months after SAH and studied their reported prevalence, pathogenesis, risk factors, clinical course and outcome.

**Results:**

We identified 16 studies describing pituitary function in the long term after SAH. The reported prevalence of endocrine dysfunction varied from 0 to 55% and the affected pituitary axes differed between studies. Due to methodological issues no inferences on risk factors, course and outcome could be made.

**Conclusions:**

Neuroendocrine dysfunction may be an important and modifiable determinant of poor functional outcome after SAH. There is an urgent need for well-designed prospective studies to more precisely assess its incidence, clinical course and effect on mood, behaviour and quality of life.

**Electronic supplementary material:**

The online version of this article (doi:10.1186/s12883-014-0205-0) contains supplementary material, which is available to authorized users.

## Background

Subarachnoid haemorrhage (SAH) accounts for 5% of stroke deaths and for more than a quarter of potential life years lost by stroke. The incidence of SAH in Western Europe is 10.5 per 100.000 persons per year and varies between regions. Case fatality ranges between 32 and 67% and about a third of patients remain dependent [1,2]. The cause of SAH is a ruptured aneurysm in 85%, perimesencephalic haemorrhage in 10%, and rare conditions in 5% of the cases [3]. Even after a good neurological recovery, a considerable proportion of patients have symptoms interfering with daily life. Fatigue, cognitive and affective dysfunction [4-7], decrease in level of activity and social participation and hence, quality of life, have been described in these patients [5]. Physical disability, social-economic status, personality, stressful events preceding SAH and life threatening illness may each contribute to the performance state of patients after SAH [4,8-11]. Glasgow Coma Scale (GCS), Hunt & Hess Scale, age, cardiac history, smoking, hypertension, new-onset seizures and mean S100B-protein levels are some of the predictors for functional outcome after SAH [12-16].

In recent years, associations have been made between SAH and hypopituitarism [17,18]. In 1914, Simmonds first described hypopituitarism as the inability of the pituitary gland to produce sufficient hormones to meet the needs of the organism. It can be caused by dysfunction of the gland itself or by an insufficient supply of hypothalamic-release-hormones. In general, hypopituitarism is a chronic condition and remains present for life [19]. Adrenocorticotropic (ACTH) and thyroid stimulating hormone (TSH) deficiency may cause fatigue, weakness, headache, altered mental activity, and impaired memory [19,20]. Growth-hormone deficiency (GHD) may cause lack of vigour, fatigue, decreased exercise tolerance and decreased social functioning [19,20]. Luteinizing hormone (LH) and follicle- stimulating hormone (FSH) deficiency in women lead to oligomenorrhea, dyspareunia, infertility and loss of libido. Testosterone deficiency in men can present with impaired sexual functioning, mood impairment, and loss of libido [19,20]. Antidiuretic hormone deficiency (ADH) leads to polyuria and polydipsia [19-21]. Many of the long-term symptoms after SAH show similarity to those occurring in patients with untreated hypopituitarism. Therefore, neuroendocrine dysfunction may be the cause or a contributing factor for residual symptoms after SAH. If this is true, deficient hormones can be supplied which may lead to improvement of these residual symptoms and improvement of long-term outcome after SAH. Nevertheless, hypopituitarism are easily overlooked after SAH and a need for routine assessment of the pituitary axes after SAH has been suggested [17].

The current review concerning neuroendocrine dysfunction in patients surviving SAH is aimed at assessing its incidence, clinical manifestation and risk factors. Furthermore, the effects of neuroendocrine dysfunction on clinical symptoms and functional outcome in patients with SAH are studied.

## Methods

### Search strategy

We searched Pubmed and Embase for articles published from 1995 to 2014 and used a combination of the term “subarachnoid haemorrhage” with “pituitary function”, “hypopituitarism”, “growth hormone”, “thyroid function”, “growth hormone”, “cortisol”, “gonadotropin”, “testosterone function” and “diabetes insipidus”. We also searched the reference lists of the articles identified by our search strategy. Two authors (LK, FK) screened titles and abstracts of all references listed in the search results independently. Of the remaining titles, full-text articles were retrieved and again screened for eligibility by both authors independently. In case of disagreement, consensus was sought through discussion. A third author (DD) was available if consensus could not be reached.

### Selection criteria

A full-text article was included in this review if it met all of the following criteria: 1) the study population consisted of patients with SAH caused by a ruptured aneurysm or, of a subgroup of patients with aneurysmal SAH; 2) the primary aim of the study was to investigate the incidence of endocrine dysfunction after SAH; 3) outcome was described in terms of levels of one or a combination of the following: ACTH, GH, TSH, cortisol, FSH, LH or testosterone, with a clear description of the assays; 4) time to laboratory investigation was at least 3 months after SAH; and 5) the study concerned a series of patients.

### Quality assessment

The two reviewers independently judged all studies by inception cohort, description of source population, description of inclusion criteria, follow-up more than 3 months, description loss to follow-up, standardized or valid measurements and data presentation of most important outcome measures. Items were scored as positive, negative or inconclusive (Table 1). A completed PRISMA checklist for quality assessment has been added as an Additional file 1. Data presented in de studies were then collected. Information on patient and study characteristics, inclusion and exclusion criteria, laboratory and outcome measurements were gathered from the selected articles.

**Table 1:** **Summary of study characteristics of studies included in this literature review**

	**Study design**	**Inclusion criteria**	**Exclusion criteria**	**Selection bias**	**Lost to FU nr**	**Dynamic tests**	**Analysis**	**Results**
Aimaretti et al. [37]	Prospective cohort	-	-	yes	0	GHRH-arg	+	+
Brandt et al. [33]	Case series	+	+	yes	0	TRH test & ITT (7 out of 10 pts)	+	+
Dimopoulou et al. [17]	Retrospective cohort	+	+	yes	0	none	+	+
Kreitschmann-Andermahr et al. [35]	Retrospective cohort	+	+	yes	10	TRH-LHRH test & ITT	+	+
Aimaretti et al. [32]	Prospective cohort	-	-	yes	0	GHRH-arg	+	+
Kreitschmann- Andermahr et al. [36]	Case series	+	+	yes	8	ITT (14 out of 45 pts)	+	+
Jovanovic et al. [34]	Case series	+	+	yes	0	none	+	+
Tanriverdi et al. [39]	Prosective cohort	+	+	no	0	GHRH-arg & glucagon test	+	+
Klose et al. [40]	Prospective cohort	+	+	no	0	ITT (GHRH-arg if contraindicated)	+	+
Lammert et al. [38]	Prospective cohort	+	+	yes	4	ACTH stimulation test (ITT in some patients)	+	+
Dutta et al. [46]	Retrospective cohort	+	+	yes	0	none	+	+
Gardner et al. [41]	Prospective cohort	+	+	no	0	GHRH-arg and glucagon test	+	+
Khursheed et al. [43]	Prospective cohort	+	+	no	0	none	+	+
Kronvall et al. [44]	Prospective cohort	+	+	no	6	GHRH-arg	no	no
Karaca et al. [45]	Prospective cohort	+	+	no	2	Glucagon test	+	+
Blijdorp et al. [42]	Prospective cohort	+	+	yes	0	Ghrelin test and GHRH-arg, Synacten test in some patients	+	+

+: inclusion criteria, exclusion criteria, assessment methods and results clearly defined and reflected, -: inclusion criteria, exclusion criteria, assessment methods and results not or not clearly defined.

GHRH- arg test: growth hormone releasing hormone plus arginine test, ITT: insulin tolerance test, d: TRH: thyrotropin releasing hormone, ACTH: adrenocorticotropic hormone, LHRH: gonadotropin releasing hormone, lost to FU number: number of patients lost in follow up of studies with more than one measurement overtime.

## Results

### Selection of studies

**Figure 1:**
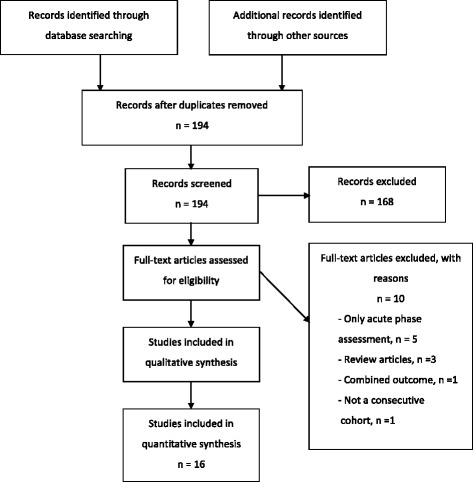
**Flowchart outlining the selection process of articles for review according to PRISMA guidelines.** From: Moher D, Liberati A, Tetzlaff J, Altman DG, The PRISMA Group (2009). Preferred Reporting Items for Systematic Reviews and Meta-Analyses: The PRISMA Statement. PLoS Med 6(6): e1000097. doi:10.1371/journal.pmed1000097. For more information, visit www.prisma-statement.org.

Initially, 194 citations (abstracts) were found. Of these citations, 62 articles did not report relevant endocrine outcome. Eighty-eight studies concerned other diseases than aneurysmal SAH. Eighteen case reports were also excluded. Twenty-six full-text articles were collected of which 5 articles reported only early phase endocrine dysfunction [22-26], 3 were review articles [27-29], 1 article reported combined data of SAH with traumatic brain injury [30] and 1 large study was excluded because it concerned an internet based data collection study [31]. Finally sixteen studies fulfilled the inclusion criteria and were eligible for the current review (Figure 1).

### Study characteristics and methodological quality

Study population size ranged from 10–93 patients. Six studies were cross-sectional or retrospective cohort studies [17,32-37]. Ten studies were conducted prospectively [37-45]. Interval between SAH and neuroendocrine assessment in studies reporting neuroendocrine outcome ranged from 3 months to 10 years.

Dimopoulou et al. retrospectively analysed 30 patients between one and two years after SAH but did not use a stimulation test for the evaluation of growth hormone function [17]. Aimaretti et al. conducted a prospective follow-up study of 40 patients after SAH derived from multiple Italian centres. The patients were all conscious and measured 3 months after discharge from the ICU. GHRH + arginine test was used to measure growth hormone function [37]. Aimaretti et al. prospectively studied 32 patients in Italian hospitals, and performed basal hormonal tests and GHRH + arginine test as a dynamic test to establish GHD between 3 and 12 months after SAH [32]. Brandt et al. selected 10 patients with fatigue after SAH and measured corticotrophin, growth hormone and thyrotrophic function using insulin tolerance test (ITT) and TSH-Thyroid releasing hormone stimulation tests12 month after SAH. In 30% of the patients ITT was not performed [33]. Kreitschman-Andermahr et al. retrospectively studied 40 SAH patients from a cohort of 274 patients after excluding patients with liver disease, coronary heart disease, convulsions, DM, depression, severe confusional state or vegetative state after discharge. ITT and THRH-LHRH were used as dynamic tests for assessment of ACTH, TSH and GH function 12 to 72 months after SAH [35]. Kreitschman-Andermahr et al. retrospectively measured basal hormones in 45 patients 3 to 24 moths after SAH. Only 14 patients had dynamic tests [36]. Jovanovic et al. retrospectively evaluated endocrine function in 93 patients, between one and ten years after SAH, however stimulation tests were not used [34]. Tanriverdi et al. prospectively analysed 22 patients one year after SAH using basal and dynamic tests for ACTH and GHD [39]. Karaca et al. did a follow-up study, three years after SAH of 20 patients investigated by Tanriverdi et al. in the abovementioned study using basal hormonal tests and glucagon stimulation test [45]. They found 4 cases of GHD three years after SAH of whom three did not have GHD one year after SAH. Dutta et al. evaluated endocrine function in 60 SAH patients with anterior communicating artery (A-com) and middle cerebral artery (MCA) aneurysms using only basal hormonal tests. Part of the study was retrospective, analysing patients one year after SAH and partly prospectively analysing patients 6 months after SAH [46]. Kronvall et al. prospectively analysed 45 patients in the acute phase and 3 to 6 months after SAH, using basal hormonal test and GHRH-arg test for GHD. They did not use a dynamic test to establish ACTH deficiency [44], Khursheed et al. prospectively analyzed 73 patients nine months after SAH for TSH and gonadotropin deficiency and not the other anterior pituitary hormones [43]. Blijdorp et al. prospectively analysed 84 patients and reported preliminary data of 43 patients using basal hormonal tests, synacten test when ACTH deficiency was suspected and a ghrelin test in the early phase after SAH and confirmatory GHRH-arg test after six months [42]. The most prominent methodological shortcomings were the incomplete reports of patient selection [17,32,33,35,37,46], selection bias [17,33-35,42,46] and inadequate laboratory testing [17,33,35,36,38,43,44]. Some studies did not use dynamic tests to determine growth hormone or corticotrophin deficiency [17,34,43,44,46]. In some reports, description of the statistical analysis and results were incomplete [36] or even absent [33].

### Frequency and type of hypopituitarism after SAH

In total, 671 patients were examined for hypopituitarism after SAH (Table 2). The proportion of patients with endocrine dysfunction varied from 0 - 55%. Growth hormone deficiency occurred in 0 to 29%, adrenocorticotropic deficiency in 0 to 40%, gonadotropin (luteinizing hormone, follicle stimulating hormone and testosterone) deficiency in 0 to 40% and thyroid stimulating hormone deficiency in 0 to 20% of patients in different studies. The largest prospective study by Klose et al. evaluated 62 patients for an average of 14 months (range 11–26 months) after SAH. Although they found some evidence of hypopituitarism after initial testing, they were not able to confirm hypopituitarism by confirmatory laboratory tests. They found no evidence of hypopituitarism in the long term after SAH [40]. In another well designed study, two different confirmatory tests were used to establish GHD adjusting the outcome for body-mass index [41]. After 12 months, 12% of the patients had pituitary dysfunction (PD).

**Table 2:** **Summary of studies assessing frequency of pituitary deficiency after SAH**

**Study**	**Time after SAH (months)**	**Patients n**	**GH%**	**ACTH%**	**TSH%**	**LH/FSH%**	**Testosterone%**	**Multiple%**	**Total%**
Aimaretti et al. [37]	3	40	25	2.5	7.5	12.5	*	10	37.5
Brandt et al. [33]	12	10	10	0	20	10	30	30	30
Dimopoulou et al. [17]	12-24	30	37	10**	7***	13	*	13	47
Kreitschmann-Andermahr et al. [35]	12-66	40	20	40	4	0	0	12	55
Aimaretti et al. [32]	12	32	21.8	6.25	9	3	3	6	37.5
Kreitschmann-Andermahr et al. [36]	3-24	45	8	13	0	0	0	9	13
Jovanovic et al. [34]	12-120	93	29	22	2.5	7.5	*	7.5	49.5
Lammert et al. [38]	6	26	0	0	4	0	0	0	4
Tanriverdi et al. [39]	12	22	36	14	0	0	0	4	50
Klose et al. [40]	12-24	62	0	0	0	0	0	0	0
Gardner et al. [41]	12	50	10	2	0	0	0	0	12
Dutta et al. [47]	6	60	15	2	13	13	23	6	31.6
Kronvall et al. [44]	3-6	45	7	18	2	4	*	NR	27
Khursheed et al. [43]	9	73	NR	NR	3	0	0	0	3
Karaca et al. [45]	36	20	20	0	0	0	0	0	20
Blijdorp et al. [42]	6	43	14	0	0	28	*	7	30

*Abbreviations*: n: numbers; %: percentage; FSH: follicle-stimulating hormone; LH: luteinizing hormone; TSH: thyroid-stimulating hormone; GH: growth hormone; ACTH: adrenocorticotropic hormone; NR: not reported; *Reporting LH, FSH and testosterone together as gonadotropin deficient; **ACTH hypo-responsive; ***Subclinical TSH deficient.

### Predictors for hypopituitarism after SAH

Inconsistent results for predicting hypopituitarism after SAH were reported. In a series of 93 patients with SAH, cerebral vasospasm and hydrocephalus were identified as risk factors for pituitary dysfunction [34]. Kreitschmann-Andermahr et al. found a significant association of female sex and the presence of corticotrophin deficiency [35]. Kronvall et al. reported that younger age was significantly associated with pituitary dysfunction at follow-up [44] but Tanriverdi et al. found higher age to be associated with growth hormone deficiency in the acute phase after SAH [39]. These findings were not confirmed by other studies [17,32,40,47].

### Association between hypopituitarism after SAH and functional recovery

In a series of 26 patients, 14 (54%) had neuropsychological deficits, but only 1 patient suffered neuroendocrine dysfunction at six months after SAH [38]. A study of 40 patients evaluated the effect of neuroendocrine dysfunction on quality of life and psychiatric symptoms. Low basal cortisol level was associated with low quality of life scores and high depression scores [48]. Severe GH deficiency was associated with low scores on the energy subscale of Nottingham Health Profile (NHP) questionnaire. Gardner et al. did not find an association between PD and quality of life after SAH, measured using the quality of life in adult GHD assessment (QOL-AGHDA) [41].

## Discussion

From the 16 studies we evaluated in this review, 15 showed some evidence for neuroendocrine dysfunction on one or more pituitary axes in the long term after SAH. In one study neuroendocrine dysfunction was only present in part of the patients in the early phase and not in the long-term after SAH. Most frequently, growth hormone deficiency was found, subsequently followed by adrenocorticotropic deficiency, gonadotropic deficiency and TSH deficiency. The reported prevalence of hypopituitarism varied from 0 to 55%. Single hormone deficiencies, mainly GHD, were more frequently found than multiple hormone deficiencies. The axes that were affected also varied among different studies.

Several mechanisms may lead to altered pituitary function in patients with SAH. Endocrine dysfunction may be provoked by compression of the hypothalamic-pituitary complex by the aneurysm itself, post-haemorrhagic local tissue pressure changes, toxic effects of extravasated blood, ischemia caused by vasospasm, increased intracranial pressure, hydrocephalus, or local destruction during craniotomy. The pituitary gland is divided into an anterior and posterior lobe. The anterior lobe is responsible for producing several peptide hormones: ACTH, TSH, prolactin, GH and gonadotropin hormones: LH and FSH. The posterior pituitary is a storage organ for the ADH and oxytocin [19]. The pituitary gland is supplied with blood from the branches of the internal carotid artery, which form a capillary plexus in the region of the median eminence of the hypothalamus. Blood from this area reaches the anterior pituitary by means of long and short portal veins through the pituitary stalk. The middle and inferior hypophyseal arteries supply the pituitary stalk and neurohypophysis with arterial blood [19]. This difference in blood supply might play a role in the pathophysiology of endocrine dysfunction after SAH, because it is the anterior pituitary hormones that are more often affected after SAH. Nevertheless, posterior pituitary can also be affected. Hyponatremia is a common symptom in the early phase of SAH [49]. The exact mechanism of this complication after SAH is still poorly understood. There are different theories about the cause of this symptom. Different study groups have suggested syndrome of inappropriate antidiuretic hormone secretion as the main cause of hyponatremia after SAH [50,51]. Yet others have suggested cerebral salt wasting syndrome due to the rise of atrial natriuretic peptide (ANP) and brain natriuretic peptide (BNP) together with volume depletion through ADH hypo-secretion [52-56]. Furthermore, due to the presence of ACTH deficiency in the early phase after SAH [25,40], ACTH deficiency has also been mentioned as one of the possible mechanisms for developing hyponatremia. Clinical evidence for this theory is lacking and needs further evaluation [51]. Intriguingly, single deficiencies were more often described than multiple hormonal deficiencies. This may imply that specific parts or systems of the anterior lobe of the pituitary gland are more vulnerable to damage than others. On the other hand, the single anterior pituitary axis deficiencies may be a marker of multiple deficiencies, which are not detected due to inappropriate testing.

The inconsistent results of the studies may be explained by differences in patient selection, time elapsed between SAH and endocrine evaluation, and different methodology of endocrine tests and definitions of hypopituitarism between the studies. Some studies were cross-sectional or retrospective studies [17,33-37,57] which are likely to be affected by selection bias, misclassification or information bias. Prospective cohort studies are best qualified in determining the occurrence of hypopituitarism after SAH. Patient selection may still be a problem in these studies [32,37,38]. There were large differences in time elapsed between SAH and the evaluation of endocrine function after SAH between studies [34,35,37,38] and even within studies [34-36]. The association between SAH and hypopituitarism could be affected by the time elapsed between SAH and the laboratory testing.

Another concern is the definition of hypopituitarism and methods used to measure endocrine function. In clinical practice and in clinical research it is difficult to define and operationalize hypopituitarism. Specific endocrine testing for each pituitary function must be performed to set an accurate diagnosis. The evaluation of pituitary function is preferably done according to an algorithm, which is interpreted by an endocrinologist in collaboration with a multidisciplinary team responsible for the patient [58]. Basal concentrations alone are not always distinctive, because of pulsatile, circadian or situational secretion. This may have led to misinterpretations of hormone values in some of the studies [17,34,46]. The assessment of ACTH and GH requires dynamic tests to reliably detect deficiency of these hormones [19]. Dynamic tests were not performed in three of the studies [17,34,46] and in some studies the tests conducted even varied within the cohort [33,36,38].

Even when dynamic tests were used validation of some of the tests such as insulin tolerance test, thyrotropin releasing hormone and adrenocorticotropic hormone stimulation test in SAH patients remains a concern [59-62]. Furthermore, the GHRH-arg test is strongly influenced by body mass index (BMI). Outcomes of GHD should be adjusted for BMI, which was not the case in various studies [32,37,39]. There is a lack of standardized tests, which makes it difficult to interpret the findings of some studies. Based on these shortcomings PD after SAH might have been over-reported in older studies [32].

Despite all shortcomings of the reviewed literature, there seems to be at least some preliminary evidence that pituitary dysfunction is associated with SAH. Younger age was associated with long-term pituitary dysfunction in one study and hydrocephalus and vasospasm was associated with pituitary dysfunction in another [34,44]. However, due to the relatively small number of patients [44] and methodological shortcomings of the studies as we mentioned earlier (case series with a time range between 1 and 10 years without pre-trial registry and no dynamic tests) [34], the role of these factors remains unclear. Still there are a few studies with a proper methodological set up and implementation but the number of cases remains small and the studies show widely diverging results [39-41].

In general, patients with hypopituitarism may have many different symptoms, including for instance fatigue, impairment of concentration, infertility, weight gain and hair loss. For clinicians, it might be efficient to use the clinical symptoms of hypopituitarism to select patients for further endocrine evaluation. However the symptoms are non-specific and do not indicate the presence or type of endocrine dysfunction accurately. In a study in which patients were selected for endocrine evaluation based on clinical symptoms of hypopituitarism [33], the reported prevalence of pituitary dysfunction was approximately 30%. This is in accordance with other studies, in which patients were not selected based on symptoms. This suggests that selection based on clinical symptoms is not efficient. On the other hand there is insufficient evidence to support routine assessment of pituitary function in all SAH patients, because the clinical relevance of pituitary dysfunction after SAH is largely unclear [63].

There were no studies reporting functional long-term outcome in association with endocrine function after SAH. In addition, we could not identify any studies that evaluated the effect of hormone substitution on any of the clinical symptoms in patients with SAH. Interestingly, there are no proper case–control designed studies answering the question whether SAH is a risk factor for the development of hypopituitarism. Such studies should have a case control design and involve a large study population, and should therefore probably be based on hospital registries. Such studies would provide a sound basis for further scientific explorations of the occurrence and risk factors for hypopituitarism after SAH.

## Conclusions

In conclusion, SAH seems to be associated with increased risk of endocrine dysfunction. Currently, there are no neurological or clinical parameters predicting the presence of hypopituitarism after SAH. Whether detection and possible treatment of endocrine dysfunction after SAH leads to better functional recovery is also unknown. Large prospective studies are needed to more precisely assess its effect on mood, behaviour and quality of life.

## Additional file

Additional file 1
**PRISMA Checklist for this review article.**
